# A potential XGBoost Diagnostic Score for *Staphylococcus aureus* bloodstream infection

**DOI:** 10.3389/fimmu.2025.1574003

**Published:** 2025-04-22

**Authors:** Junhong Shi, Lan Chen, Xinru Yuan, Jinjin Yang, Yanlei Xu, Li Shen, Yu Huang, Bingjie Wang, Fangyou Yu

**Affiliations:** ^1^ Department of Clinical Laboratory, Shanghai Pulmonary Hospital, School of Medicine, Tongji University, Shanghai, China; ^2^ Department of Clinical Laboratory Medicine, The First Affiliated Hospital of Ningbo University, Ningbo, Zhejiang, China; ^3^ Department of Respiratory Medicine, The First Affiliated Hospital of Wenzhou Medical University, Wenzhou, China

**Keywords:** *Staphylococcus aureus*, bloodstream infection, WGCNA, Diagnostic Score, XGBoost

## Abstract

*Staphylococcus aureus* (*S. aureus*) bloodstream infection is often life-threatening, and increasing in incidence. We identified 63 differentially expressed genes (DEGs) in the GSE33341 *S. aureus* infection samples. Subsequently, intersecting the 63 DEGs with 950 genes from the blue module through weighted gene co-expression network analysis (WGCNA) yielded 38 genes. We leveraged Boruta and least absolute shrinkage and selection operator (LASSO) algorithms and identified5 diagnostic genes (DRAM1, UPP1, IL18RAP, CLEC4A, and PGLYRP1). Comparative analysis revealed that Extreme Gradient Boosting (XGBoost) surpassed SVM-RFE and Random Forest models, demonstrating superior diagnostic performance for *S. aureus* bloodstream infection (mean AUC for XGBoost =0.954; mean AUC for SVM-RFE =0.93275; mean AUC for Random Forest =0.94625). The XGBoost Diagnostic Score correlated with multiple immune cells to varying degrees, manifesting significant negative associations with CD8 T cells and CD4 naive T cells in both human and mouse samples. The diagnostic power of the Diagnostic Score was further validated by RT-qPCR results obtained from both mouse and patient samples, as well as RNA-Seq analysis conducted on mouse samples. XGBoost Diagnostic Score, consisting of DRAM1, UPP1, IL18RAP, CLEC4A, and PGLYRP1, may serve as a Diagnostic tool for *S. aureus* bloodstream infection.

## Introduction

*Staphylococcus aureus* (*S. aureus*) was a common pathogen causing both community and hospital infections (1, 2). *S. aureus* can lead to acute and chronic infections in various parts of the body, including the bloodstream, respiratory tract, bone marrow, and other parts of the body (3–5). Among these, bloodstream infection was particularly harmful and had a higher incidence rate (6–8). Clinically, *S. aureus* bloodstream infection is defined clinically by positive *S. aureus* blood cultures from patients exhibiting signs and symptoms of systemic infection (9).

Once *S. aureus* entered the bloodstream, it triggered the recruitment of circulating neutrophils and the production of inflammatory cytokines (10, 11). CD4 naive T cells proliferated strongly in response to superantigens such as staphylococcal enterotoxin B (SEB) (12). Superantigens are bacterial virulence factors that induce a state of immune hyperactivation by forming a bridge between certain subsets of T cell receptor (TCR) β chains on T lymphocytes, and class II major histocompatibility complex (MHC-II) molecules (13, 14). Zhang et al. found that in the face of superantigens induced immune hyperactivation, an intact bacterium-specific CD4+ T cell response can be mounted (15). Cytotoxic CD8+ T cells eliminated target cells by recognizing homologous epitopes presented by MHC class I (MHC-I) molecules, playing a role in clearing intracellular bacteria in murine models of bacterial infection (16–18). Alexeev confirmed that cytotoxic CD8+ T cells recognized *S. aureus*-infected Recessive dystrophic epidermolysis bullosa keratinocytes and responded by producing interleukin-2 (IL-2) and IFNγ and degranulating and cytotoxically killing infected cells (19). Yi et al. discovered that the potential T-cell antigens of *S. aureus*, EsxA and EsxB, can play a critical role in stimulating T helper 1 immunity by activating IgG2a and CD8(+) T cells (20). *S. aureus* can exhibit a high bacterial burden during endovascular infection through foci formation that adhere to vessel walls (3). Therefore, the internalization and the persistence of *S. aureus* within host cells allowed the bacteria to evade these responses.

Methods for identifying disease-associated modules and genes were increasingly being developed, which can be effective in searching for diagnostic markers in clinical practice (21, 22). Diagnostic biomarkers are defined as indicators that can detect or confirm the presence of a disease or condition, or identify individuals with specific disease subtypes (23). These biomarkers serve not only to diagnose patients but also to refine the classification and understanding of diseases. Recent research into diagnostic biomarkers has increasingly emphasized the usage of advanced methodologies to boost predictive accuracy and improve patient outcomes. For instance, weighted gene co-expression network analysis (WGCNA) was widely recognized as a robust method for uncovering relationships between genes and clinical features (24–26). On top of that, Extreme Gradient Boosting (XGBoost) was efficient, flexible, and lightweight, and has been widely used in data mining, recommender systems, and other fields (27). Among various machine learning algorithms, XGBoost has demonstrated superior performance in medical applications. Recent studies have indicated that the XGBoost algorithm exhibits enhanced predictive power compared to alternative algorithms. For instance, Shen et al. developed an XGBoost-based machine learning model that showed superior performance compared with the other three machine learning algorithms in predicting lobar pneumonia in children with Community-acquired pneumonia (28). In addition, among the seven machine learning predictive models incorporating non-high-density lipoprotein cholesterol to high-density lipoprotein cholesterol ratio, the XGBoost algorithm exhibited the highest predictive performance, with an area under the curve (AUC) of 0.828 (29).

In conclusion, this study comprehensively investigated the predictive diagnosis of *S. aureus* bloodstream infection deploying an ensemble machine-learning approach. The Diagnostic Score, constructed by combining the biomarkers DRAM1, UPP1, IL18RAP, CLEC4A, and PGLYRP1 harnessing the XGBoost algorithm, demonstrated superior diagnostic performance. Bioinformatic analysis combined with experimental validation provided a solid foundation for predicting *S. aureus* bloodstream infection. These findings were anticipated to offer new approaches for the diagnosis and treatment of *S. aureus* bloodstream infection.

## Methods

### Public gene expression datasets

Access to the Gene Expression Omnibus (GEO) database (https://www.ncbi.nlm.nih.gov/geo/), a public repository of high-throughput gene expression data, chips, and microarrays, was adopted for data collection. We queried the GEO database with the keywords “*Staphylococcus aureus*” [MeSH Terms] and “Bloodstream infection” [All Fields]. None of the included samples were concerned with any other diseases. The sample size of both the pediatric sepsis group and the normal group was greater than 10. Ultimately, GSE33341-human was selected as the training dataset, and GSE33341-mice served as the test dataset (30). Datasets GSE65088 (31) and GSE16129 (32) were employed for validation.

### Weighted correlation network analysis

WGCNA is a genomics research method that facilitates the identification of gene clusters with high relatedness (24, 33, 34). This approach constructs a coexpression network using the “WGCNA” R package, focusing on the top 7500 genes exhibiting the highest variance. The network enables systematic gene screening to identify potential biomarkers or therapeutic targets. Hierarchical clustering identifies modules of genes according to their expression patterns, grouping those with similar patterns into distinct modules. This process categorizes tens of thousands of genes into multiple modules, adopting correlation and correlation coefficients as key measures.

### Evaluation of functional enrichment

Gene Ontology (GO) and the Kyoto Encyclopedia of Genes and Genomes (KEGG) pathway enrichment analyses were conducted via the “clusterProfiler” (35) package in R to explore possible biological features of blue modules. Additionally, Gene set enrichment analysis (GSEA) (36) was performed via the “clusterProfiler” package in R to investigate the enrichment pathways.

### Identification of the differentially expressed genes

The Wilcoxon test was utilized to identify differentially expressed genes (DEGs) between the *S. aureus* bloodstream infection group and the control group in the GSE33341 dataset. A P-value < 0.05 and |log2FC| > 1 were set as the cutoffs for DEGs.

### Construction of diagnostic models for *S. aureus* bloodstream infection

Firstly, up-regulated genes in the human and mice *S. aureus* infection groups from GSE33341 were intersected to acquire 63 DEGs associated with *S. aureus* bloodstream infection. Afterward, these genes intersected with 950 genes from the blue module identified by WGCNA, resulting in 38 genes. Boruta and least absolute shrinkage and selection operator (LASSO) logistic regression were then applied separately to identify biomarkers, ultimately selecting five genes. Boruta algorithm is a full correlation analysis screening feature algorithm for identifying and selecting the set of features that are relevant to the dependent variable. It can select the essential features from the original feature set while excluding the features that do not have a significant impact on the model predictions (37). LASSO logistic regression is a penalized regression method that forces some coefficients to zero, effectively reducing variables and improving model achievement when the number of predictors exceeds the sample size (38). Diagnostic Score models were constructed by deploying these five genes through XGBoost, Support Vector Machine-Recursive Feature Elimination (SVM-RFE), and Random Forest (RF) algorithms. XGBoost is a decision-tree-based ensemble algorithm that ameliorates model performance by optimizing the loss function through gradient boosting and introducing regularization to improve generalization (39), with the following hyperparameters: eta = 0.01, max_depth = 2, min_child_weight = 3, subsample = 0.5, colsample_bytree = 0.6. SVM-RFE is a boundary-based supervised learning algorithm effective for classification tasks by constructing hyperplanes in high-dimensional space to maximize the margin between classes (40). RF is an ensemble learning technique that improves predictive accomplishment by constructing multiple decision trees and integrating their outcomes (41). The predictive power of the Diagnostic Score was evaluated using the receiver operating characteristic (ROC) curve and calculating the area under the curve (AUC). After comparing the mean AUC in four datasets, the XGBoost model (mean AUC for XGBoost =0.954; mean AUC for SVM-RFE =0.93275; mean AUC for Random Forest =0.94625) (mean accuracy for XGBoost =0.84; mean accuracy for SVM-RFE =0.7825; mean accuracy for Random Forest =0.7425), which demonstrated the best performance, was selected for in-depth analysis.

We utilized SHAP values to assess the overall feature importance in the XGBoost model. SHAP, a recent advancement in making tree-based models more interpretable, employs a game-theoretic method that aggregates the local contributions of individual features to explain the model’s behavior on a global scale. This approach is considered superior to other global approximation methods. The algorithm not only provides a measure of feature importance across the model but also offers insights into the role of each feature in specific predictions.

Sensitivity is the proportion of true positives tests out of all patients with a condition (42). In other words, it is the ability of a test or instrument to yield a positive result for a subject that has that disease. The ability to correctly classify a test is essential, and the equation for sensitivity is the following: Sensitivity=(True Positives (A))/(True Positives (A)+False Negatives (C)).

Specificity is the percentage of true negatives out of all subjects who do not have a disease or condition. In other words, it is the ability of the test or instrument to obtain normal range or negative results for a person who does not have a disease (43). The formula to determine specificity is the following: Specificity=(True Negatives (D))/(True Negatives (D)+False Positives (B)).

### Assessment of immune cell infiltration

Immune cell infiltration was assessed by computing the differential infiltration of 22 immune cells exploiting the CIBERSORT (44) algorithm in the human samples and 36 immune cells using the ImmuCellAI (45) algorithm in the mouse samples. These algorithms are computational methods that depend on gene expression data to estimate the relative abundance of various cell types within a sample. The correlation between gene expression and immune cells was evaluated using Pearson’s correlation coefficients, with correlation plots produced utilizing the “ggpubr” R package.

### Clinical blood samples

Whole blood samples were collected from five patients diagnosed with *S. aureus* bloodstream infection and from five healthy controls at the Shanghai Pulmonary Hospital, School of Medicine, Tongji University, Shanghai, China, for RNA extraction and subsequent RT-qPCR analysis. The blood samples used in this study were by-products of routine care in the department of clinical laboratory.

### Establishment of a mouse model for *S aureus* bloodstream infection

The Methicillin-sensitive *Staphylococcus aureus* (MSSA) Newman strain was cultured for 16 hours in TSB medium at 37°C. Overnight cultures were centrifuged at 2683g (RCF) for 5 minutes at room temperature and secondarily adjusted to a concentration of 2 × 10^9^ CFU/mL with the help of phosphate-buffered saline (PBS). Succeeding this, female Balb/c mice were injected via the tail vein with 100 μL PBS containing 2 × 10^8^ CFU bacterial cells suspended. At eight hours post-infection, the mice were anesthetized with 2,2,2-tribromoethanol (5 mg/25 g). Surgical scissors were operated to remove mouse whiskers; thereafter, tweezers were leveraged to clamp the eyeball and swiftly excise it, allowing blood to flow from the eye socket into the EP tube. The collected blood sample was immediately placed in liquid nitrogen and stored at −80°C until RNA extraction.

### RNA extraction and real-time polymerase chain reaction

Total RNA from patients and mice blood samples were isolated using the RNA Prep Pure Blood kit (Tiangen Biotech Co.; Ltd.) following the manufacturer’s protocol. RNA was then reverse transcribed into cDNA utilizing the PrimeScript RT reagent kit with gDNA Eraser (Takara). Real-time quantitative PCR (RT-qPCR) was performed using TB GreenTM Premix Ex TaqTM II (Takara) on QuantStudioTM 5 Real-Time PCR System (Applied Biosystems). The expression levels of DRAM1, UPP1, IL18RAP, CLEC4A, and PGLYRP1 genes normalized to GAPDH were calculated using the formula 2^−ΔΔCt^. All primers used in this study were listed in **Supplementary Table S1**. Each reaction was performed thrice.

### Statistical analysis

All statistical analyses and graphical representations were conducted using R version 4.3.1 except for RT-qPCR results which were analyzed through GraphPad Prism version 9 (GraphPad Software Inc. San Diego, CA, USA). Statistical significance was determined by a two-tailed test with a P value of less than 0.05. *P < 0.05, **P < 0.01, ****P < 0.0001.

## Results

### Establishment of WGCNA and detection of key modules

**Figure 1:**
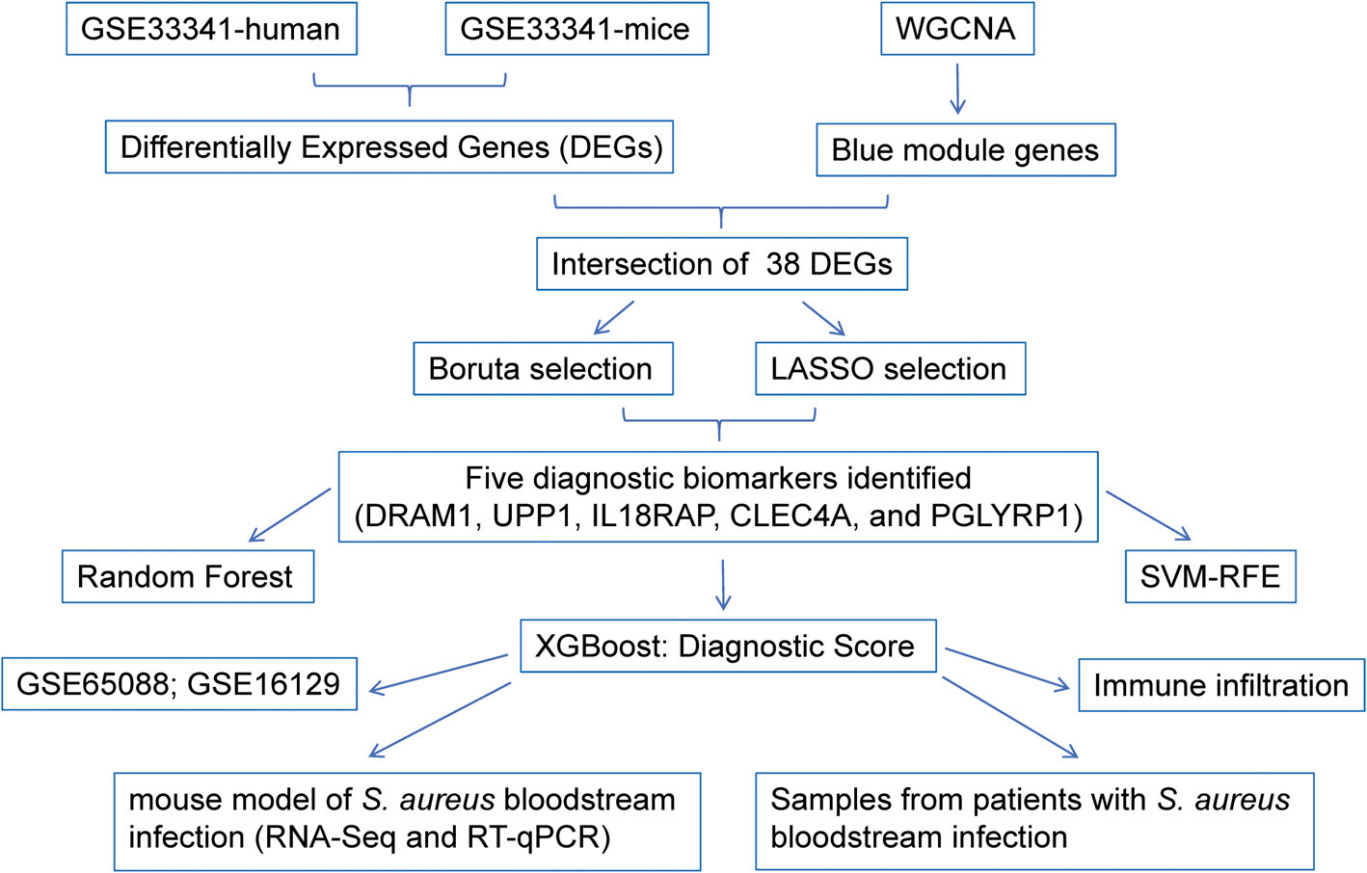
Schematic presentation of the analysis process.

**Figure 2:**
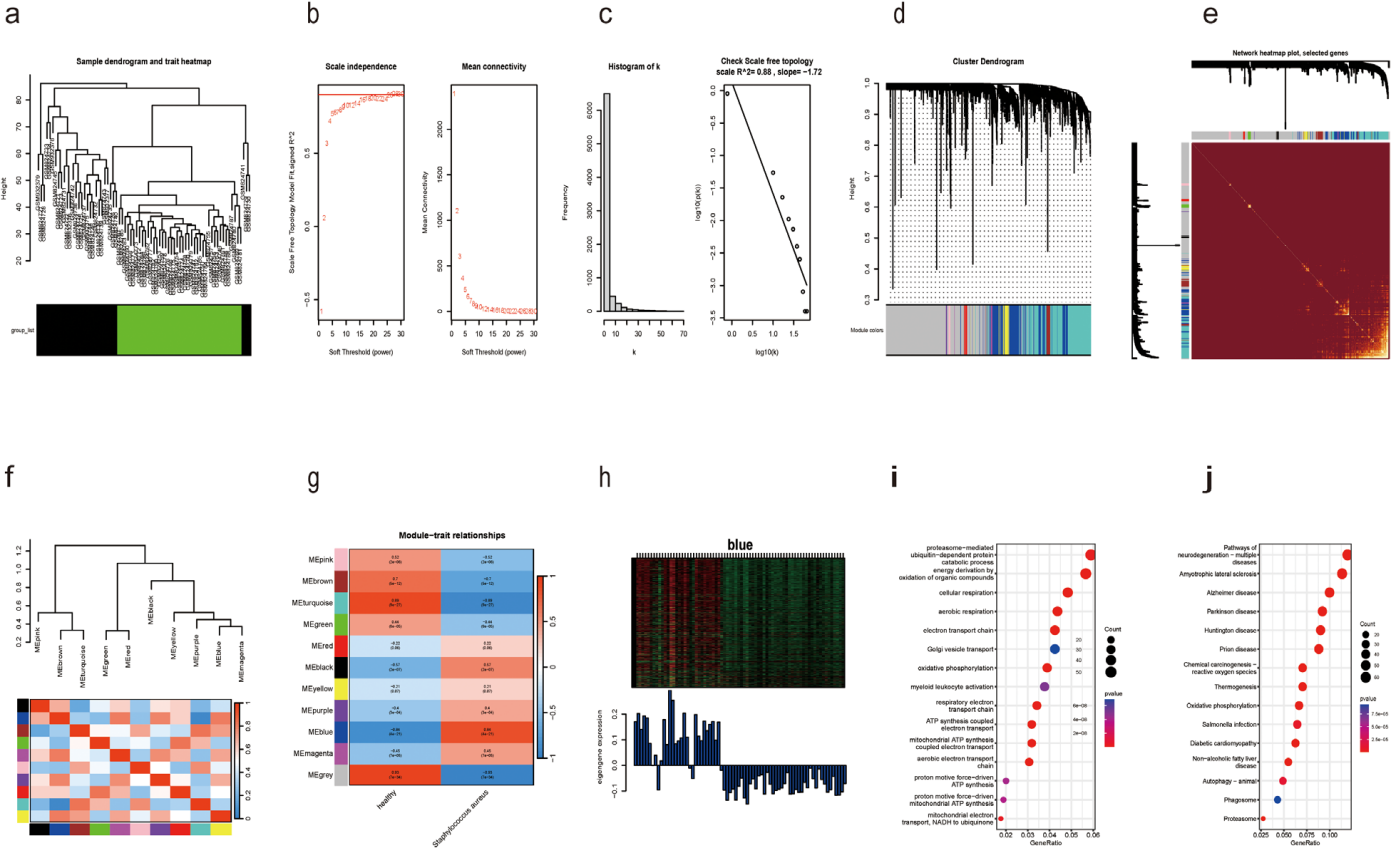
Identification of modules associated with *S. aureus* bloodstream infection using WGCNA. **(a)** Dendrogram of sample clustering; **(b)** Soft threshold selection in the WGCNA. Scale independence and mean connectivity were used for soft threshold selection in WGCNA; **(c)** Distribution of nodes with the degree of connection, (k) Correlation of log (k) and log [P(k)]; **(d)** Hierarchical cluster trees showing coexpression modules identified by WGCNA; **(e)** Interaction relationship analysis of coexpressed genes. Different colors of the horizontal and vertical axes represent different modules. The brightness line in the middle represents the degree of connectivity of different modules; **(f)** Hierarchical clustering dendrogram of module eigengenes with color labels; **(g)** Module–trait relationships. Each cell contains the corresponding correlation and p-value. The table is color-coded by correlation according to the color legend; **(h)** Expression heatmap of the blue module and feature vector gene histogram of the blue module; **(i)** GO analysis of the enriched genes in the blue module; **(j)** The KEGG pathways of the enriched genes in the blue module.

**Figure 1** illustrated the research design of this study. PRISMA methodology was utilized for extensive identification of public datasets (**Supplementary Figure S1**). To precisely identify the central genes connected with the *S. aureus* infection phenotype, we constructed a gene co-expression network adopting the WGCNA algorithm. The results from hierarchical clustering analysis demonstrated robust clustering among the samples, with no significant outliers observed (**Figure 2A**). The trends in Scale Independence and Mean Connectivity as functions of Soft Threshold (power) are illustrated in **Figure 2B**. The construction of the final scale-free network was depicted in **Figure 2C**, where the left panel presented a histogram of network connectivity while the right panel displayed a log-log plot corresponding to this histogram. A high R² value of 0.88 indicated an approximate scale-free topology (**Figure 2C**). A gene hierarchy clustering dendrogram was generated following gene correlations, resulting in the identification of eleven similar gene modules (**Figure 2D**). Branches of the dendrogram (the modules) were grouped together based on the correlation of eigengenes (**Figure 2E**). Interaction connections among the eleven modules were analyzed, leading to the creation of a network heatmap presented in **Figure 2F**. As portrayed in **Figure 2G**, there existed a highest positive correlation between the blue module and *S. aureus* infection, implying that this module may foster disease progression related to *S. aureus* infection. Furthermore, **Figure 2H** illustrated that expression levels of feature vector genes ME showed a strong correlation with expressions within all genes comprising this module; thus, expression values for characteristic genes can be considered representative profiles for their respective modules. Given that the blue module correlates positively with *S. aureus* infection, its constituent genes were selected for subsequent KEGG and GO analyses GO enrichment analysis revealed that genes within blue modules were enriched in processes such as proteasome-mediated Ubiquitin-dependent protein, catabolic process energy derivation by oxidation of organic compounds, and cellular respiration (**Figure 2I**). Likewise, KEGG analysis for genes in blue modules was associated with Pathways related to neurodegeneration-multiple diseases, Amyotrophic lateral sclerosis, Alzheimer disease, Parkinson disease, and Chemical carcinogenesis-reactive oxygen species (**Figure 2J**).

### The diagnostic score of *S. aureus* bloodstream infection constructed by XGBoost exhibited the most superior diagnostic capacity

**Figure 3:**
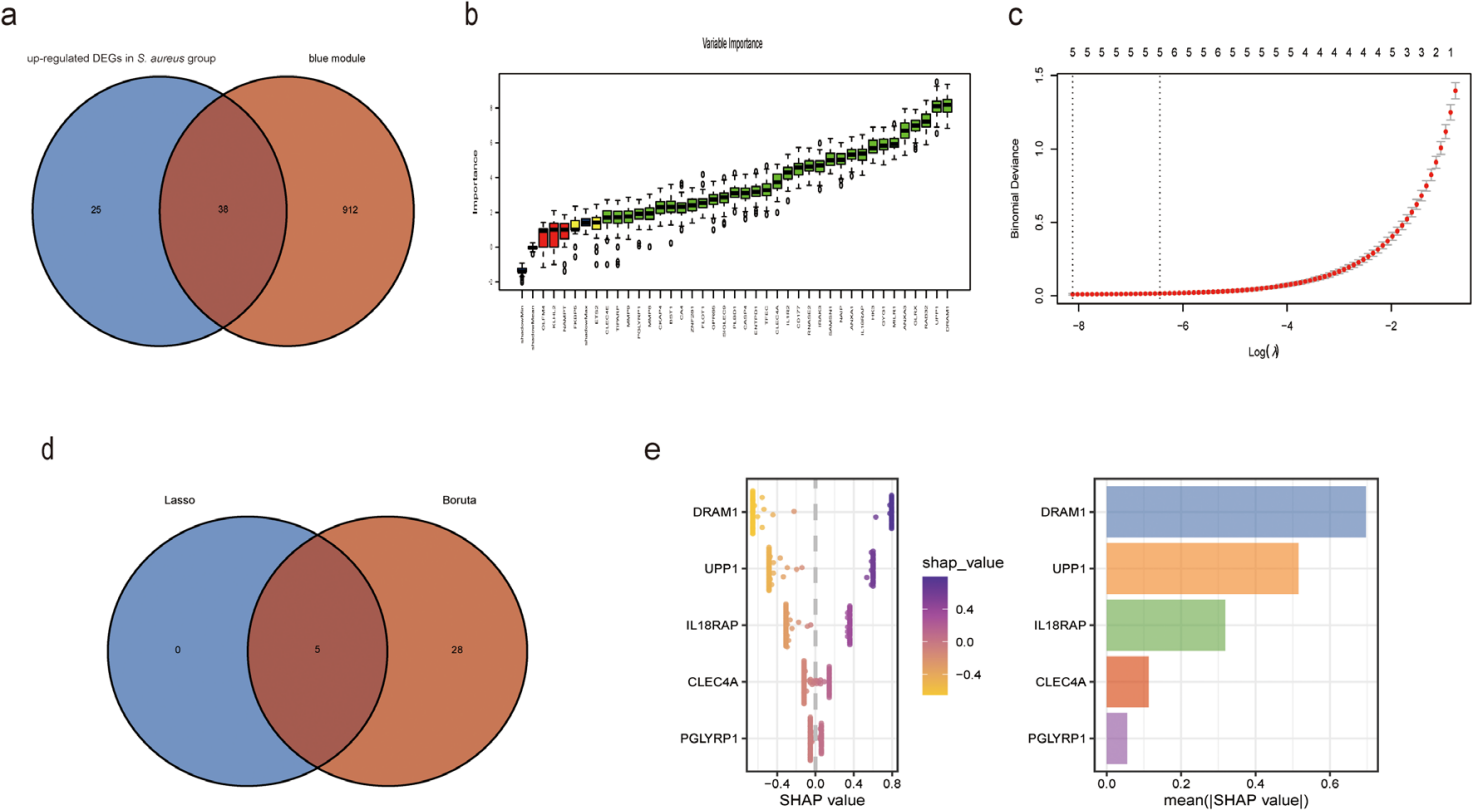
XGBoost algorithm was employed to generate the Diagnostic Score for *S. aureus* bloodstream infection. **(a)** Venn plot of the intersected genes that were obtained by DEGs in *S. aureus* bloodstream group and blue module; **(b)** Based on Boruta to screen biomarkers; **(c)** Based on LASSO regression analysis to screen biomarkers; **(d)** Venn diagram showing the intersection of diagnostic markers obtained by the two algorithms; **(e)** In accordance with their mean absolute values, SHAP beeswarm plot for the five features demonstrate the relationship between these features and S. aureus bloodstream infection. Each point represents one patient. High values of the features were shown in red and low values were shown inblue. SHAP analysis exhibits the average impact on model output.

The clinical characteristics of the two sample groups are detailed in **Supplementary Table S2**. Human blood samples from GSE33341 included 31 *S. aureus* infection samples and 43 control samples; mouse blood samples from GSE33341 comprised 103 *S. aureus* infection samples and 64 control samples. The up-regulated genes in the human and mice *S. aureus* infection groups facilitated the identification of 63 DEGs. An intersection of these DEGs with the 950 genes identified in the blue module via WGCNA yielded a total of 38 overlapping genes (**Figure 3A**). We further employed Boruta and LASSO algorithms to identify feature genes (**Figures 3B, C**). Eventually, both algorithms pinpointed DRAM1, UPP1, IL18RAP, CLEC4A, and PGLYRP1 as potential diagnostic markers (**Figures 3D, E**).

**Figure 4:**
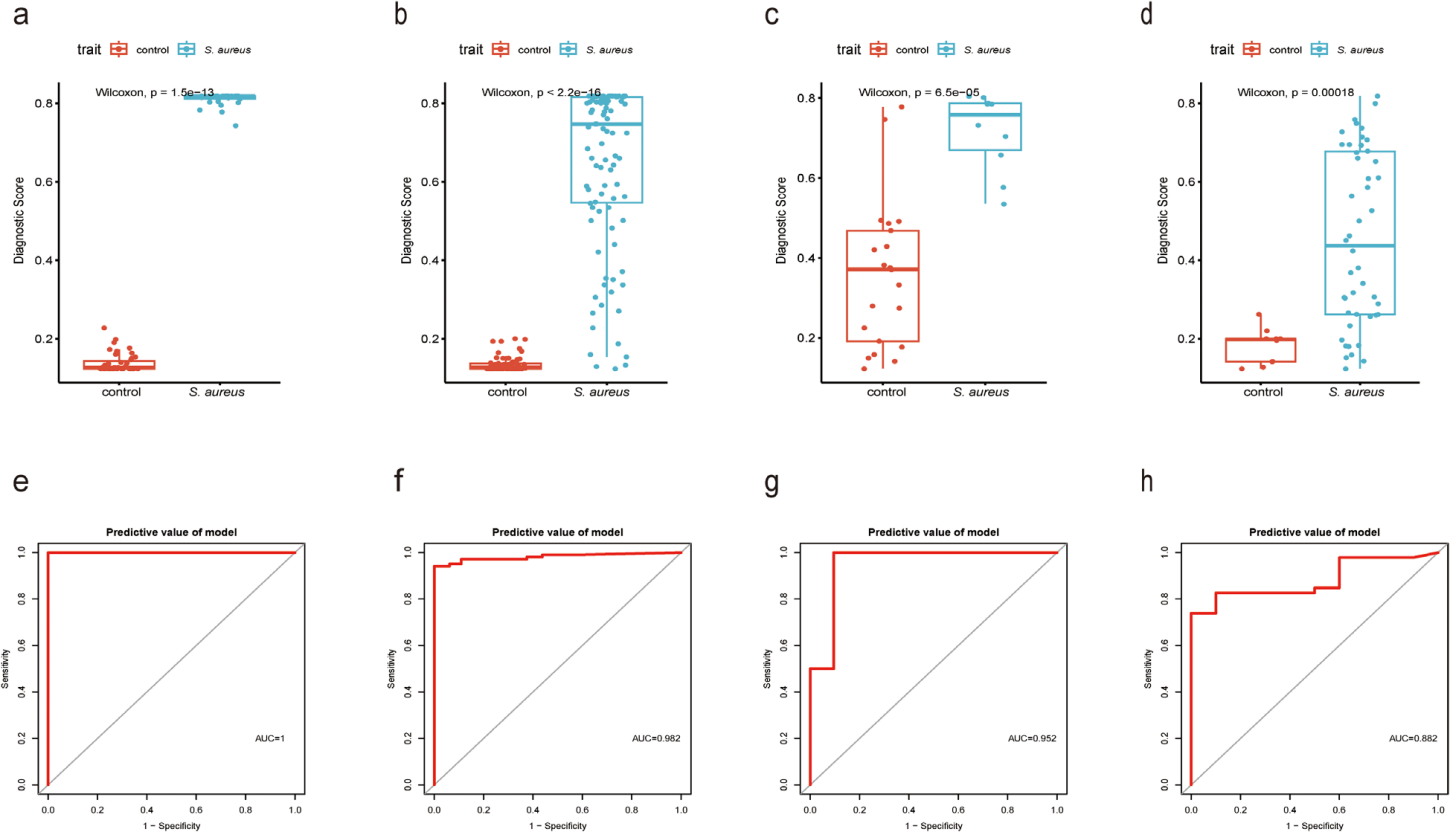
Performance of XGBoost evaluation on training and validation datasets. **(a)** Boxplot showed the expression of Diagnostic Score between the *S. aureus* infection group and control group in GSE33341 human group by XGBoost; **(b)** Boxplot showed the expression of Diagnostic Score between the *S. aureus* infection group and control group in GSE33341 mice group by XGBoost; **(c)** Boxplot showed the expression of Diagnostic Score between the *S. aureus* infection group and control group in GSE65088 by XGBoost; **(d)** Boxplot showed the expression of Diagnostic Score between the *S. aureus* infection group and control group in GSE16129 by XGBoost; **(e)** The ROC curve of the diagnostic efficacy verification of Diagnostic Score between the *S. aureus* infection group and control group in GSE33341 human group by XGBoost; **(f)** The ROC curve of the diagnostic efficacy verification of Diagnostic Score between the *S. aureus* infection group and control group in GSE33341 mice group by XGBoost; **(g)** The ROC curve of the diagnostic efficacy verification of Diagnostic Score between the *S. aureus* infection group and control group in GSE65088 by XGBoost; **(h)** The ROC curve of the diagnostic efficacy verification of Diagnostic Score between the *S. aureus* infection group and control group in GSE16129 by XGBoost.

**Figure 5:**
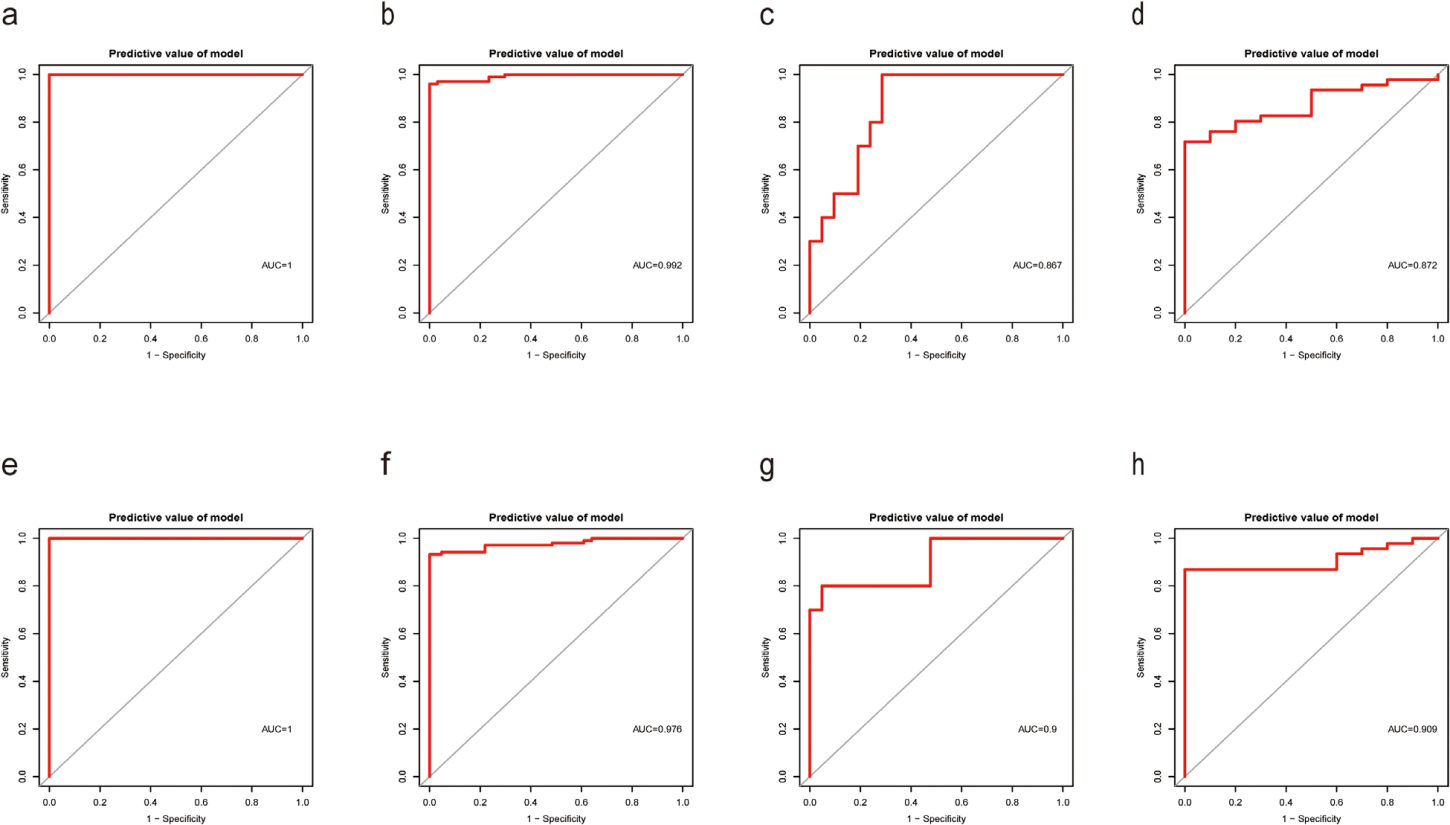
Performance of SVM-RFE and RF models on training and validation datasets. **(a)** The ROC curve of the diagnostic efficacy verification of Diagnostic Score between the *S. aureus* infection group and control group in GSE33341 human group by SVM-RFE; **(b)** The ROC curve of the diagnostic efficacy verification of Diagnostic Score between the *S. aureus* infection group and control group in GSE33341 mice group by SVM-RFE; **(c)** The ROC curve of the diagnostic efficacy verification of Diagnostic Score between the *S. aureus* infection group and control group in GSE65088 by SVM-RFE; **(d)** The ROC curve of the diagnostic efficacy verification of Diagnostic Score between the *S. aureus* infection group and control group in GSE16129 by SVM-RFE; **(e)** The ROC curve of the diagnostic efficacy verification of Diagnostic Score between the *S. aureus* infection group and control group in GSE33341 human group by RF; **(f)** The ROC curve of the diagnostic efficacy verification of Diagnostic Score between the *S. aureus* infection group and control group in GSE33341 mice group by RF; **(g)** The ROC curve of the diagnostic efficacy verification of Diagnostic Score between the *S. aureus* infection group and control group in GSE65088 by RF; **(h)** The ROC curve of the diagnostic efficacy verification of Diagnostic Score between the *S. aureus* infection group and control group in GSE16129 by RF.

To originate a Diagnostic Score for *S. aureus* bloodstream infection utilizing these five identified genes, we applied XGBoost alongside SVM-RFE and RF algorithms. In **Figures 4E-H** and **Figure 5**, ROC curves derived from four datasets were presented separately for XGBoost algorithm, SVM-RFE, and RF analyses; notably, the XGBoost algorithm exhibited the highest mean AUC values (mean AUC for XGBoost =0.954; mean AUC for SVM-RFE =0.93275; mean AUC for RF =0.94625). Consequently, we confirmed that the diagnostic model constructed by XGBoost demonstrated exceptional diagnostic capability regarding *S. aureus* bloodstream infection.

In addition, in the GSE33341 human and mouse groups, the Diagnostic Score was not only highly expressed in the *S. aureus* infection group (**Figures 4A, B**) but also demonstrated good diagnostic efficacy between *S. aureus* and the control group (**Figures 4E, F**). Besides, GSE65088 and GSE16129 were acquired to validate the above findings. A significantly elevated Diagnostic Score in the *S. aureus* group was observed (**Figures 4C, D**), accompanied by high AUC values (**Figures 4G, H**), indicating that the Diagnostic Score possessed considerable predictive power.

### Assessment of immune cell infiltration

To evaluate the difference in immune cell infiltration between the *S. aureus* infection group and the control group, we availed the CIBERSORT algorithm to calculate the infiltration of 22 immune cell types for human samples from GSE33341, while using ImmuCellAI to analyze the infiltration of 36 immune cells for mouse samples from GSE33341.

**Figure 6:**
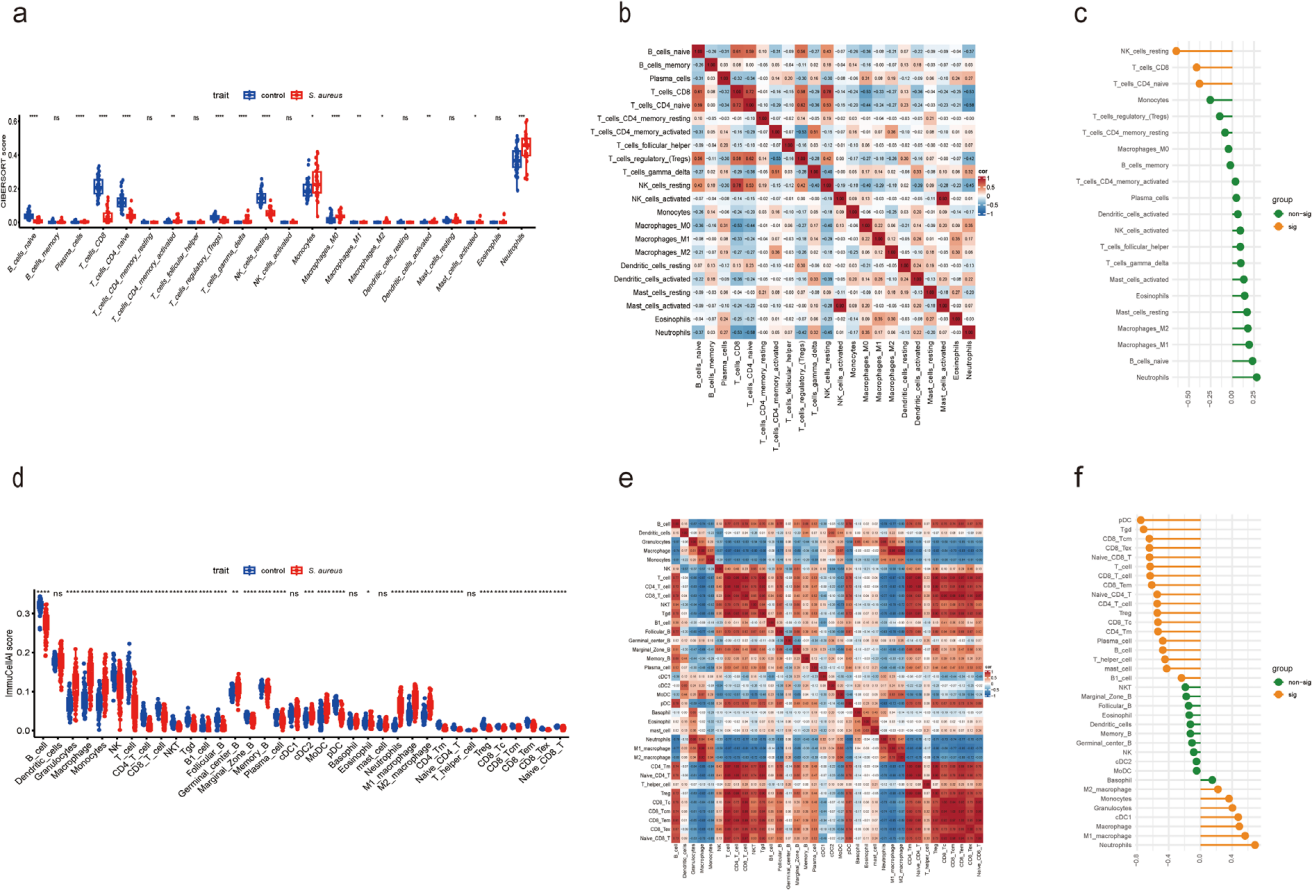
Compared and correlated in immune cell infiltration. **(a)** Boxplot showed the distinct fractions compared to each immune cell between the *S. aureus* and control group in GSE33341 human samples. **(b)** Analysis of correlations among 22 immune cell subtypes in GSE33341 human samples. The horizontal and vertical axes represented the names of immune cells, and the values stood for the correlation coefficients between immune cells. Red denoted a positive correlation, while blue denoted a negative correlation. **(c)** Correlation between Diagnostic Score and immune cells in GSE33341 human samples. **(d)** Boxplot showed the distinct fractions compared to each immune cell between the *S. aureus* and control group in GSE33341 mouse samples. **(e)** Analysis of correlations among 36 immune cell subtypes in GSE33341 mouse samples. The horizontal and vertical axes represented the names of immune cells, and the values stood for the correlation coefficients between immune cells. Red denoted a positive correlation, while blue denoted a negative correlation. **(f)** Correlation between Diagnostic Score and immune cells in GSE33341 mouse samples. *P < 0.05, **P < 0.01, ***P < 0.001, ****P < 0.0001, ns, not significant.

Boxplots were constructed to represent each sample based on distinct subpopulation infiltrations The boxplots revealed significant infiltration of monocytes, neutrophils, and M2 macrophages within both human and mouse samples from the *S. aureus* group. In contrast, higher infiltration of CD4 naive T cells, CD8 T cells, and Tregs was noted in both human and mouse control groups (**Figures 6A, D**). For human samples from GSE33341, the analysis implied that activated NK cells exhibited a strong positive relevance with activated mast cells; nevertheless, CD4 naive T cells displayed a notable negative association with neutrophils (**Figure 6B**). Similarly, relationships among immune cells derived from mouse samples highlighted that CD4 T cells showed strong positive correlations with both CD4 Tm cells as well as CD4 naive T cells; conversely, macrophages exhibited a marked negative association with T cell populations (**Figure 6E**).

By coincidence, we ascertained that the Diagnostic Score was significantly negatively germane to CD8 T cells and CD4 naive T cells in human samples from GSE33341 (**Figure 6C**), which was highly consistent with the results in mouse samples from GSE33341 (**Figure 6F**). Meanwhile, the human data manifested a notable negative correlation between the Diagnostic Score and resting NK cells as well. In addition, our analysis reflected that the Diagnostic Score for mouse samples was notably positively correlated with neutrophils, macrophages, cDC1, granulocytes, and monocytes. However, there was a significant negative association between the Diagnostic Score and various immune cell types including pDCs, Tgd cells, B cells, T cells, Tregs, as well as both CD8 Tcm and naive CD8 T cells (**Figure 6F**).

The aforementioned analytical results suggest that the Diagnostic Score was significantly negatively associated with both CD8 T cells and CD4 naive T cells across human and mouse samples. The development of immunological prophylaxis and therapy targeting *S. aureus* represents an appealing objective. Our findings endorsed a pragmatic application of the Diagnostic Score for identifying potential patients at risk for *S. aureus* bloodstream infection who may benefit from immunotherapy. The relationship between these genes and immune infiltrating cells underscores their potential role in the pathogenesis of *S. aureus* bloodstream infection while offering new insights for early detection and treatment strategies.

### Validation for the expression difference and diagnostic value of the diagnostic score by a mouse model and patient samples

**Figure 7:**
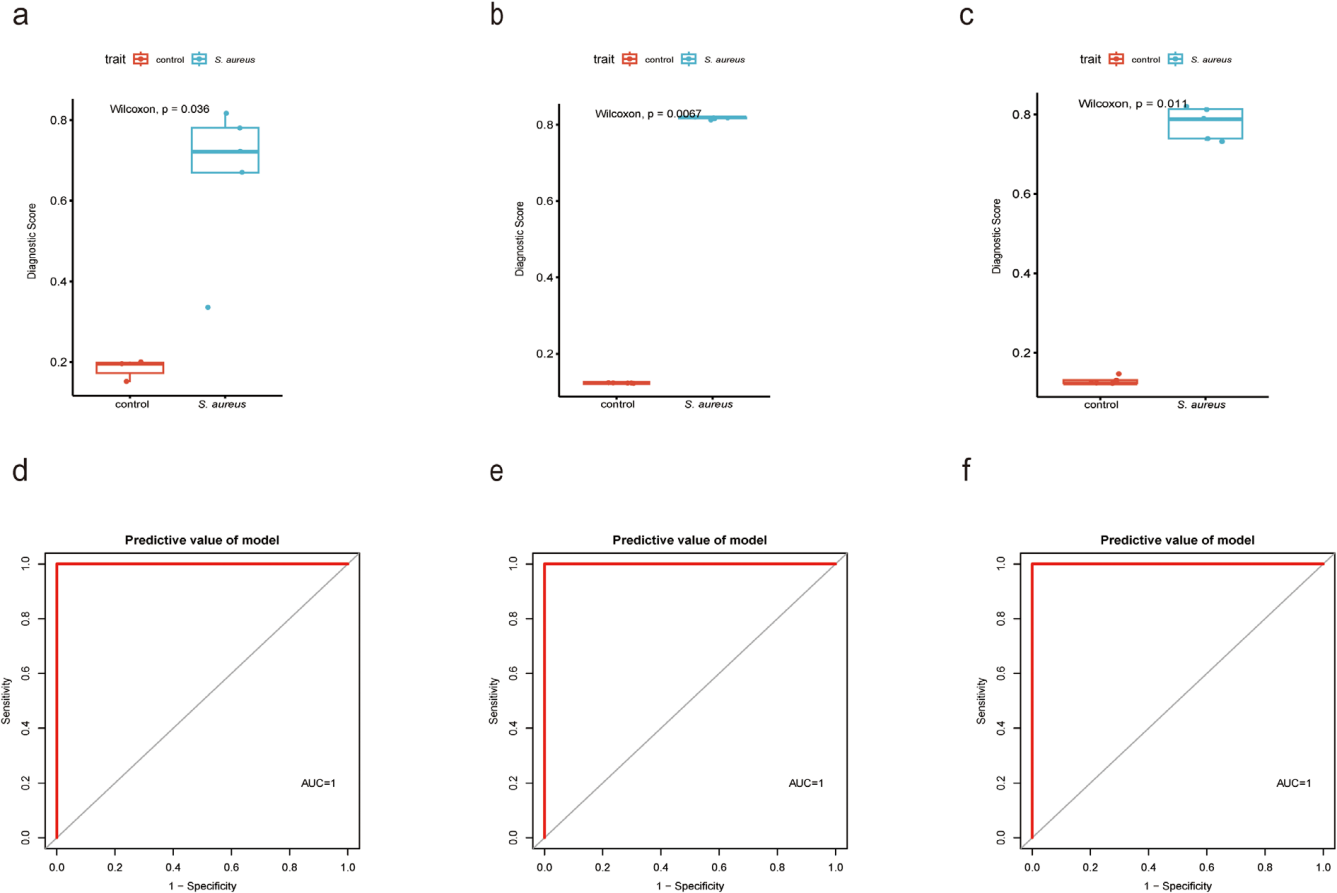
Validation for the diagnostic value of the Diagnostic Score by a mouse model and patient samples. **(a)** Boxplot showed the expression of Diagnostic Score between the *S. aureus* infection group and control group in RNAseq analysis by XGBoost; **(b)** Boxplot showed the expression of Diagnostic Score between the *S. aureus* infection group and control group in RT-qPCR results from mouse samples by XGBoost; **(c)** Boxplot showed the expression of Diagnostic Score between the *S. aureus* infection group and control group in RT-qPCR results from patients samples by XGBoost; **(d)** The ROC curve of the diagnostic efficacy verification of Diagnostic Score between the *S. aureus* infection group and control group in RNAseq analysis by XGBoost. **(e)** The ROC curve of the diagnostic efficacy verification of Diagnostic Score between the *S. aureus* infection group and control group in RT-qPCR results from mouse samples by XGBoost; **(f)** The ROC curve of the diagnostic efficacy verification of Diagnostic Score between the *S. aureus* infection group and control group in RT-qPCR results from patients samples by XGBoost.

To further validate the diagnostic utility of the Diagnostic Score, we established a mouse model of *S. aureus* bloodstream infection and collected blood samples for RNA-Seq analysis in our previous study (46)and RT-qPCR experiments. The choice of the *S. aureus* Newman strain was based on its characterization as a hypervirulent strain that has been extensively utilized in various models involving *S. aureus* infections. The results from RNA-Seq analysis corroborated our previous findings (**Figure 7A**), with ROC curve analyses indicating a high diagnostic value for the Diagnostic Score (**Figure 7D**). Furthermore, RT-qPCR results proved that the expression levels associated with the Diagnostic Score were significantly elevated in the *S. aureus* Newman treatment group compared to those in the control group (**Figure 7B**), thereby reinforcing its diagnostic significance (**Figure 7E**). Most importantly, the analysis of clinical samples collected by our team confirmed that Diagnostic Score was significantly overexpressed in patients with *S. aureus* bloodstream infection (**Figure 7C**), demonstrating high diagnostic value (**Figure 7F**).

## Discussion

*S. aureus* is a prevalent bacterial pathogen responsible for bloodstream infection, with an annual incidence ranging from 12 to 18 cases per 100,000 populations (47–49). Despite a decline in the case fatality rate associated with *S. aureus* bloodstream infection over the past three decades, this rate remains alarmingly high at approximately 27% within three months post-infection (50–52). In the realm of sepsis management, advanced machine-learning approaches have been shown to significantly improve the accuracy of short-term outcome predictions, such as 30-day mortality rates, thereby underscoring their potential advantages over traditional models (53). To provide more comprehensive diagnostic information and optimize diagnostic precision, this study harnessed machine-learning integration methods aimed at optimizing predictive model presentation through the utilization of multiple algorithms.

To accurately identify key genes associated with the *S. aureus* infection phenotype, we constructed a gene co-expression network adopting the WGCNA algorithm. By intersecting genes from the blue module identified through WGCNA screening with up-regulated DEGs in *S. aureus* infection samples, we obtained a total of 38 candidate genes. Subsequently, two algorithms including Boruta and LASSO regression analysis, were employed to pinpoint DRAM1, UPP1, IL18RAP, CLEC4A, and PGLYRP1 as potential diagnostic markers. Most importantly, we applied XGBoost alongside SVM-RFE and RF algorithms to derive Diagnostic Scores for *S. aureus* bloodstream infection based on these five identified genes. Comparative analyses revealed that XGBoost outperformed other models and exhibited exceptional diagnostic capabilities for detecting *S. aureus* bloodstream infection. To conclude, we designated the XGBoost algorithm as instrumental in defining five genes as part of our proposed Diagnostic Score for *S. aureus* bloodstream infection. In summary, the XGBoost Diagnostic Score represents a novel approach to diagnosing *S. aureus* bloodstream infection that holds considerable clinical promise.

In recent years, many studies in the field of bacterial infection immunology have focused on the five genes mentioned above. DNA damage-regulated autophagy regulator 1 (DRAM1) is a stress-induced regulator that regulates autophagy and cell death (54). A study published in *Nature* suggested that DRAM1 was relevant to interferon-induced expression signature in tuberculosis patients (55). Vaart et al. confirmed that DRAM1 linked mycobacterial recognition via TLR-MYD88 to autophagic defense (56). Uridine phosphorylase 1 (UPP1) encodes uridine phosphorylase, a key enzyme that regulates intracellular uridine and 2’-deoxyuridine metabolism (57). Research conducted by Hamasaki et al. revealed that UPP1 was upregulated in all three brain regions of sepsis-associated encephalopathy samples (58). Moreover, Fan et al. identified UPP1 as a notable diagnostic marker for pediatric septic shock (59). Interleukin 18 receptor accessory protein (IL18RAP) encodes the accessory subunit of the heterodimeric receptor for interleukin 18 (IL18), a proinflammatory cytokine involved in inducing cell-mediated immunity (60). This protein enhances the IL18-binding activity of the IL18 receptor and plays a role in signaling by IL18 (61). Chen et al. disclosed that IL18RAP might be closely linked with *Escherichia coli* (*E. coli*)-induced sepsis (62). A study conducted by Vaher pointed out that the NLRP1-dependent inflammasome could be activated in the skin by *S. aureus*, which may lead to increased levels of the IL-18 and IL-1b cytokines in the skin and thereby contribute to the development of atopic dermatitis, especially in more susceptible individuals with respective sequence variants in the IL18RAP gene (63). Apart from that, C-type lectin domain family 4 member A (CLEC4A) encodes a member of the C-type lectin/C-type lectin-like domain (CTL/CTLD) superfamily. Members of this family share a common protein fold and have diverse functions, such as cell adhesion, cell-cell signaling, glycoprotein turnover, and roles in inflammation and immune response (64). Zhang et al. identified CLEC4A as a potential prognostic marker for Chronic lymphocytic leukemia (65). The research of Uto et al. supported that CLEC4A was a potential target for immune checkpoint blockade in tumor immunotherapy (64). Peptidoglycan recognition protein 1 (PGLYRP1) Enables several functions, including Hsp70 protein binding activity; peptidoglycan binding activity; and peptidoglycan immune receptor activity (66). Involved in antimicrobial humoral immune response mediated by antimicrobial peptide and defense response to Gram-positive bacterium (67, 68). Chen et al. confirmed that the expression of PGLYRP1 protein in *S. aureus*-induced mastitis tissues was significantly higher than that in healthy tissues (69). Gong et al. identified PGLYRP1 as critical in sepsis and sepsis-related Acute respiratory distress syndrome (ARDS) by bioinformatic analysis (70). Zhang et al. found that PGLYRP1 was upregulated, which may result in dysfunction of immune response in sepsis (71). The above results suggested the importance of these five genes in the field of bacterial infection. Similarly, the high expression levels associated with the Diagnostic Score constructed by these five genes were validated in our mouse bloodstream infection model and clinical patient samples, demonstrating that these five genes have good diagnostic value for *S. aureus* bloodstream infection.

Neutrophils are key players in the host defense against *S. aureus*. These cells circulate in the bloodstream and are recruited to tissues by locally produced chemoattractants following *S. aureus* infection (72). Although CD4 T naive cells do not directly participate in bacterial killing, they can promote *S. aureus* clearance by increasing the recruitment of phagocytic cells to the site of infection and by augmenting their antimicrobial activity via the production of cytokines (73, 74). During *S. aureus* skin infections, the bacterium stimulates the proliferation of CD8+ cytotoxic T lymphocytes (CTLs) via activation of LN-resident dendritic cells (75). Immune infiltration analysis indicated significant alterations in the immune microenvironment between *S. aureus* and healthy groups. Notably, the Diagnostic Score was discovered to be significantly negatively correlated with both CD8 T cells and naive CD4 T cells across human and mouse samples. Therefore, the Diagnostic Score had the potential as a biomarker for predicting responses to immunotherapy, facilitating personalized treatment strategies that mitigate unnecessary burdens on patients due to inappropriate therapies. In spite of that, this study had certain limitations. Although we have collected clinical samples from patients with *S. aureus* bloodstream infection for verification, the number of samples was not large enough, and more samples need to be collected for validation. Furthermore, animal and cellular models will be established to investigate the molecular mechanisms underlying *S. aureus* induced bloodstream infection in greater depth.

In summary, this study thoroughly investigated predictive diagnostics for *S. aureus* bloodstream infection using an integrated machine-learning approach. The findings accentuated that the Diagnostic Score derived from the XGBoost algorithm, incorporating five genes (DRAM1, UPP1, IL18RAP, CLEC4A, and PGLYRP1), showed promising diagnostic efficiency for bloodstream infection caused by *S. aureus*. These findings promise novel avenues for diagnosing and treating *S. aureus* bloodstream infection while contributing significantly to early diagnosis and intervention efforts aimed at improving patient outcomes through targeted therapeutic approaches.

## Data Availability

The datasets analyzed during this study are available in the GEO database (https://www.ncbi.nlm.nih.gov/geo) (GSE33341, GSE65088, and GSE16129). Further inquiries can be directed to the corresponding author.

## Supplementary material

Supplementary Figure 1 PRISMA workflow for datasets used in this study.

Supplementary Figure 2 Validation of diagnostic markers using RT-qPCR. **(a-e)** Expression of five genes in patients with *S. aureus* bloodstream infection; **(f-j)** Expression of five genes in a mouse model of *S. aureus* bloodstream infection. *P < 0.05, **P < 0.01, ****P < 0.0001.
