# Supplementary figures and images for: A potential XGBoost Diagnostic Score for Staphylococcus aureus bloodstream infection
Source: Front Immunol. 2025 Apr 22;16:1574003. doi: 10.3389/fimmu.2025.1574003 (PMC12052945; doi:10.3389/fimmu.2025.1574003)

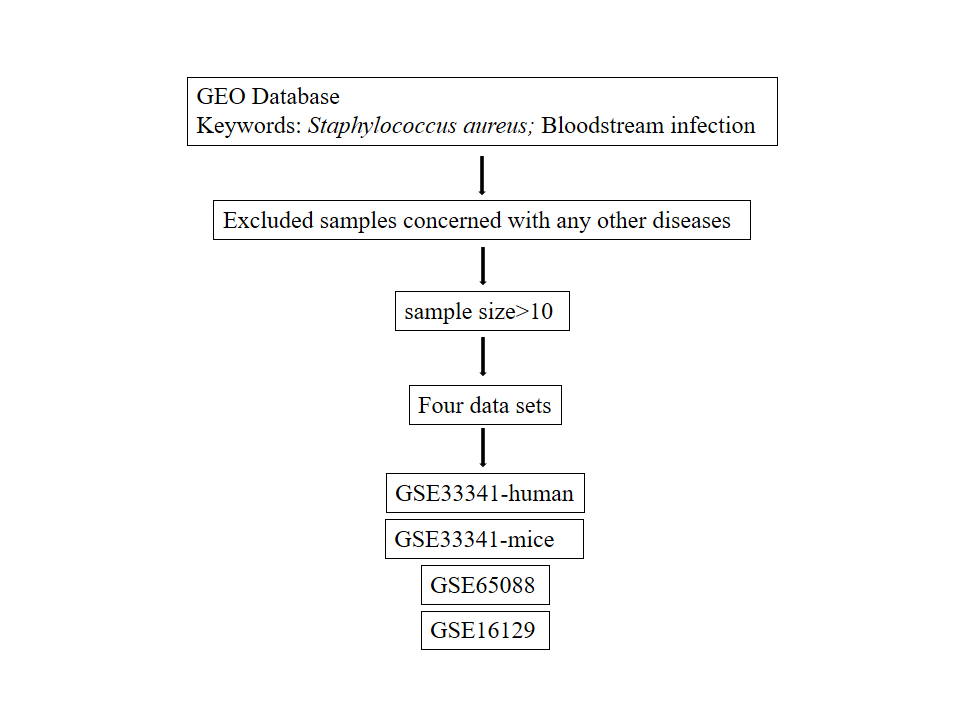

Supplement: Supplementary Figure 1 — PRISMA workflow for datasets used in this study. [file Image1.tif]

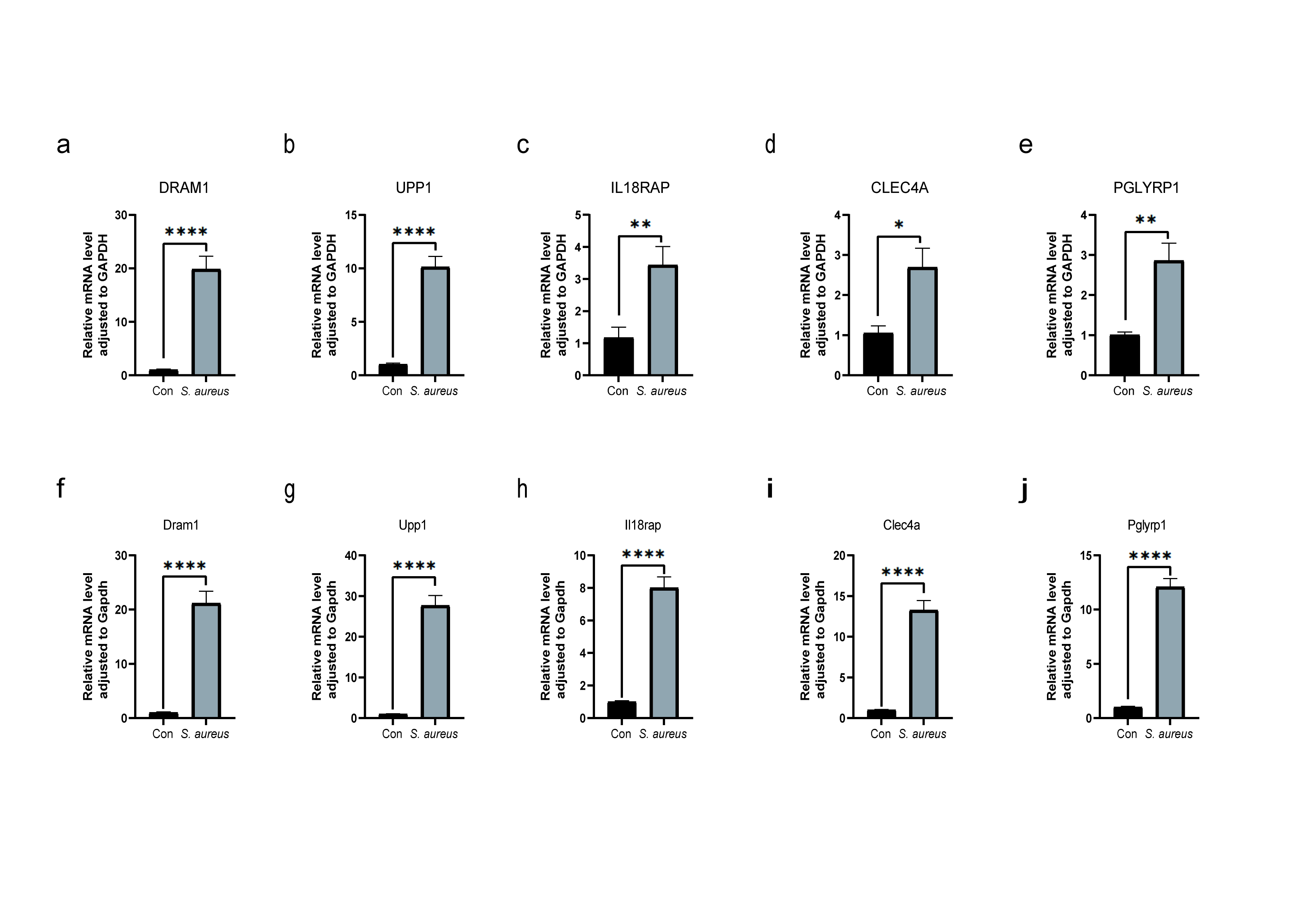

Supplement: Supplementary Figure 2 — Validation of diagnostic markers using RT-qPCR. (a-e) Expression of five genes in patients with S. aureus bloodstream infection; (f-j) Expression of five genes in a mouse model of S. aureus bloodstream infection. *P < 0.05, **P < 0.01, ****P < 0.0001. [file Image2.tif]
