# Supplementary material for: A potential XGBoost Diagnostic Score for Staphylococcus aureus bloodstream infection
Source: Front Immunol. 2025 Apr 22;16:1574003. doi: 10.3389/fimmu.2025.1574003 (PMC12052945; doi:10.3389/fimmu.2025.1574003)
Supplement: Supplementary file 3 [file Table1.docx]

| **Primer** | **Primer sequence (5’→3’)** |
| --- | --- |
| Mouse-Dram1-F | CATCTCCGCTGTTTCGTGC |
| Mouse-Dram1-R | GGATTCCATTCCAGCTTGGTTA |
| Mouse-Upp1-F | CGAAGTGATTGACTGGTGGT |
| Mouse-Upp1-R | TTCAGTTCCTGTAGCTGCCA |
| Mouse-Il18rap-F | CCTTGTCAAAAAGGCTCTGC |
| Mouse-Il18rap-R | CCAGGGGTTTCTGTGTCACT |
| Mouse-Clec4a-F | GCCCAAAGGATTGGAGGCTA |
| Mouse-Clec4a-R | CTGCTCTTCCTGGCTTTGGA |
| Mouse-Pglyrp1-F | GCAATGTGCAGCATTACCAC |
| Mouse-Pglyrp1-R | CTGTGTGGTCACCCTTGATG |
| Mouse-Gapdh-F | AGGTCGGTGTGAACGGATTTG |
| Mouse-Gapdh-R | TGTAGACCATGTAGTTGAGGTCA |
| Human-DRAM1-F | TTGGCCTTTGTCTGTGGTGT |
| Human-DRAM1-R | TCCGTATGTGGCATGTCGAG |
| Human-UPP1-F | CACCACTAGCAGACACAATTTCC |
| Human-UPP1-R | AATGCCCATACCATGACTGACAG |
| Human-IL18RAP-F | TGAAGA ACACTTGGCCCTGA |
| Human-IL18RAP-R | GCAAGATTCACTGCTGCTTGT |
| Human-CLEC4A-F | CTTGGCAAGACAGTGAGAAGGAC |
| Human-CLEC4A-R | TGACCTTCTGGATCTGAGAGCC |
| Human-PGLYRP1-F | AGCGGCTCAGGAGACAGAAGAC |
| Human-PGLYRP1-R | ATAGCGTAAGGGCAGGCTCAGG |
| Human-GAPDH-F | GCACCGTCAAGGCTGAGAAC |
| Human-GAPDH-R | TGGTGAAGACGCCAGTGGAC |

Primer pairs used in RT-PCRs.
