# Supplementary material for: A potential XGBoost Diagnostic Score for Staphylococcus aureus bloodstream infection
Source: Front Immunol. 2025 Apr 22;16:1574003. doi: 10.3389/fimmu.2025.1574003 (PMC12052945; doi:10.3389/fimmu.2025.1574003)
Supplement: Supplementary file 4 [file Table2.docx]

| **GEO dataset** | **Platform** | 1. **aureus infection** | **Healthy** |
| --- | --- | --- | --- |
| GSE33341-human | GPL570 | 31 | 43 |
| GSE33341-mice | GPL1261 | 103 | 64 |
| GSE65088 | GPL10558 | 10 | 21 |
| GSE16129 | GPL6106 | 46 | 10 |

Information on microarray datasets obtained from GEO.
